# Peer Review of “The Impact of SARS-CoV-2 Lineages (Variants) and COVID-19 Vaccination on the COVID-19 Epidemic in South Africa: Regression Study”

**DOI:** 10.2196/46908

**Published:** 2023-07-03

**Keywords:** COVID-19, infection, pandemic, vaccine, vaccination, epidemiology, transmissibility, health care, hospital admission, COVID-19 variants, SARS-CoV-2

*This is a peer-review report submitted for the paper “The Impact of SARS-CoV-2 Lineages (Variants) and COVID-19 Vaccination on the COVID-19 Epidemic in South Africa: Regression Study.”*

## Round 1 Review

### General Comments

This paper [1] discusses the impact of the SARS-CoV-2 lineage in the South African COVID-19 epidemiology because it is important to investigate the evolution of distinct SARS-CoV-2 lineage that dominates among three epidemic waves in South Africa. The authors begin by recalling the background of the COVID-19 global pandemic and introducing the SARS-CoV-2 lineage and its variants. In section 2, their methodology is introduced. The data were obtained from public sources. Descriptive statistics, paired sample *t* test, and regression analysis with new variables such as active cases, deaths, and daily patient discharge are provided. The authors interpret the results of statistical analyses and discuss their findings from the data in section 3. However, the manuscript should be polished. Here are some comments.

### Specific Comments

#### Major Comments

1. Throughout the manuscript, the notation of numbers is not consistent. For example, in the middle of the second paragraph in section 1, Introduction, “The genome of SARS-CoV-2 is a single positive-stranded RNA approximately 29 903 bases (nucleotides) pairs in length 9 [2-5].” It looks like a space between numbers indicates a digit of a thousand, and a comma is omitted. However, in the middle of the paragraph in section 2.2.1., “Table 2 shows that the mean COVID-19 daily tests in the first, second and third South African COVID-19 epidemic wave period were 20 575±14 062, 31 046±14 115 and 46 822±18 460 respectively.” A space between numbers indicates a decimal point, not a comma.

2. Sections 2 and 3 are extremely difficult to read because they are too lengthy, although subsections indicate each statistical analysis that was performed. I believe that the authors do not need to provide outputs copied from SPSS directly. Are all columns in each table meaningful? Should readers know both standard deviation and variance for each statistic, for example? I strongly suggest that the authors get rid of unnecessary columns in each table and move unnecessary tables from sections 2 and 3 to the appendix.

3. I believe that the *P* values in the manuscript do not need to be specific. For example, Table 3 displays Pearson and Spearman correlation coefficients and *P* values. Many people may not understand what 9.94E-79 means. It can be simplified to “<0.001” or 0.

#### Minor Comments

4. The font style and size are not consistent throughout the manuscript.

## Round 2 Review

### General Comments

The authors have tried to improve the quality of the manuscript. However, the manuscript still needs substantial improvement. Please see my comments.

### Specific Comments

#### Major Comments

1. This issue has not been resolved. The authors said that the space between numbers indicates a digit of a thousand. However, according to JMIR house style and editorial guidelines, numbers greater than 999 have a comma to separate thousands, millions, etc. Please see [6] and update the style of numbers throughout the manuscript.

2. The authors have reduced unnecessary columns. However, the JMIR production team suggests no more than 5 tables per manuscript. There are still unnecessary tables in the manuscript, that do not provide meaningful information and are just the same outputs of SPSS. What is the purpose of including so many tables without interpretation? Should Table 1 really be placed in the main manuscript? Why? Please see [7].

3. The authors have updated the representation of *P* values according to the suggestion of the editorial director [8].

4. The font style is still not consistent throughout the manuscript. Please revise the font style.

5. The Introduction in the manuscript is too long. I would suggest reducing the Introduction in the manuscript.

6. There are 13 equations in the manuscript. I believe that the authors can reduce the number of equations in the manuscript by combining similar equations. Listing all equations is unnecessary. Also, reference numbers for equations could be a number in the parenthesis such as (1) instead of Equation 1.

7. Detailed information about the paired test (what pairs to what) will be placed in the footnote in the corresponding table or figure.

8. Why do the authors think that the following text or Table 3 is needed in the manuscript?

“Table 3 shows that the Pearson (Spearman) Correlation Coefficients between COVID-19 daily tests (Independent Variable) and cases (Dependent Variable) in the first, second, third and fourth COVID-19 epidemic wave in South Africa were 0.910 (0.955), 0.877 (0.751), 0.893 (0.847) and 0.854 (0.812) respectively.”

This text and Table 3 are the same information.

9. What is the reason to provide Pearson correlation and Spearman rho together? Do the authors want to show a linear relationship or an ordinal relationship?

#### Minor Comments

10. The footnotes in Tables 3 and 4 are redundant. Where are the superscripts a, b, or c in the tables?

11. There is an inconsistent number of digits in all tables in the manuscript.

12. From Tables 1 to 16, why do the authors think that the minimum and maximum provide meaningful information in 2?

13. Please use “95% confidence interval” instead of “95 % confidence interval.”

## Round 3 Review

### General Comments

The authors have improved the manuscript’s quality compared to the previous version. However, I would assume that the quality could be improved more if the authors addressed the following comments.

### Major Comments

1. In “Covariance and Regression of South African Epidemiological Data,” the authors stated that the 2-tailed Pearson correlation above 0.850 with *P*<.001 was considered as having a high degree of linearity. Pearson correlation coefficient has a value between –1 and 1. A negative value (eg, –0.850) could also be considered as a strong negative relationship between two variables. Was a negative relationship included in the determination of linearity?

2. In “Normalisation and Paired T-tests on South African Epidemiological Data,” the authors considered only 7 pairs among 5 periods. Normalized parameter 2 and 4, normalized parameter 2 and 5, and normalized parameter 3 and 5 were not included in pairing. Was there a specific reason to exclude these three pairs in the paired *t* test?

3. In the Discussion, the authors stated that the Pfizer-BioNTech (Comirnaty) and the Johnson & Johnson/Janssen COVID-19 vaccines have shown high efficacy against severe COVID-19 at 85% and 88.9%, respectively. However, two terms, vaccine efficacy and effectiveness, are used in different settings. According to [9], Pfizer demonstrated their COVID-19 vaccine efficacy based on randomized controlled trials. However, Johnson & Johnson did not show their COVID-19 vaccine efficacy according to [10]. Instead, Johnson & Johnson demonstrated their COVID-19 vaccine effectiveness based on observational studies, which is in a real-world setting. Could you please clarify this? (Please see [11].)

### Minor Comments

4. The authors did not explain what the special characters after SARS-CoV-2 variants mean (eg, BA.4# or BA.2.75***). Could you please provide details on what the special characters after SARS-CoV-2 variants indicate?

5. The authors used unnecessary abbreviations throughout the manuscript. Could you please review the manuscript and remove some unnecessary abbreviations that are not used in a section of the manuscript?

## Round 4 Review

### Specific Comments

#### Major Comments

1. It is difficult to understand what Tables 2 and 3 show. Table 3 provides the mean difference between two daily positive COVID-19 tests in a percentage. If we look at the paired differences mean of pair 5 (daily positive COVID-19 test 2 – daily positive COVID-19 test 3), the difference is –1.20. However, the mean of the daily positive COVID-19 test 2 is 11.5 and the mean of the daily positive COVID-19 test 3 is 13.3 in Table 2. Could you please clarify what you compare between the two groups? How do we understand Tables 2 and 3 together? The same comment will be applied to Tables 4 and 5.

#### Minor Comments

2. The notation of *P* values throughout the manuscript is inconsistent.

On page 5, “with Pearson correlations above 0.850 or below -0.850 with *P*<.001 considered as having a high degree of linearity.” On page 8, “The Spearman’s correlation coefficients and P-values between the daily cumulative COVID-19 vaccinated people and the daily COVID-19 cases in the first half period of the third, fourth and fifth COVID-19 epidemic wave in South Africa were 0.930 (95% CI 0.890-0.956), 0.842 (95% CI 0.713-0.916) and 0.811 (95% CI 0.673-0.895) respectively with *P*-values<.001. While the Spearman’s correlation coefficients and P-values between the daily cumulative COVID-19 vaccinated people and the change in daily COVID-19 cases were 0.031 (*P*=.79 95% CI -0.207-0.266), -0.014 (*P*=.93 95% CI -0.341-0.316) and -0.077 (*P*=.62 95% CI -0.374-0.233) respectively.” Could you please make an update on the notation?

## Round 5 Review

### General Comments

The authors’ responses are clear. However, this paper still needs cosmetic improvement. I have some minor comments to improve the quality of this manuscript.

### Specific Comments

#### Minor Comments

1. In Tables 1 and 2, some minimum values are “-.” Does this mean zero or unknown? Could you please specify what “-” is?

2. The format of *P* values in Table 3 and the tables in the appendix is incorrect. Please edit based on [8].

3. Tables 6, 7, and 8 show both standard deviation and variance. Are there any specific reasons that the authors display both? If there is no reason, it is sufficient to show the standard deviation only.
